# Suicidality during treatment with serotonin and norepinephrine reuptake inhibitors

**DOI:** 10.1192/j.eurpsy.2024.396

**Published:** 2024-08-27

**Authors:** I. Da Fonseca Pinto, A. Elias de Sousa, M. A. Vieira-Coelho

**Affiliations:** ^1^Department of Psychiatry and Mental Health, University Hospital Center of São João; ^2^Department of Biomedicine - Pharmacology and Therapeutics Unit, Faculty of Medicine of Porto University, Porto, Portugal

## Abstract

**Introduction:**

Treatment choice when prescribing antidepressants for major depressive disorder (MDD) is often influenced by safety and tolerability profiles. A transient increase in suicidality following antidepressant treatment initiation is a key concern. Although rare, its unpredictability and consequences make them a significant worry. In 2004, the U.S. Food and Drug Administration (FDA) issued a “black-box” warning regarding a potential increase in suicidality in adolescents receiving antidepressant treatment for depression that was later expanded to include both young adults and a broader range of antidepressants.

**Objectives:**

The aim of this study is to evaluate the risk of increased suicidality during the treatment with serotonin and norepinephrine reuptake inhibitors (SNRIs) in young adults with MDD.

**Methods:**

We conducted a non-systematic literature search on PubMed using the combination of MeSH terms ([Serotonin and Noradrenaline Reuptake Inhibitors] OR [Levomilnacipran] OR [Desvenlafaxine Succinate] OR [Venlafaxine Hydrochloride] OR [Duloxetine Hydrochloride]) AND [Suicide] AND [Young Adult], and the keywords [(“Serotonin and Noradrenaline Reuptake Inhibitors” OR “Levomilnacipran” OR “Desvenlafaxine” OR “Venlafaxine” OR “Duloxetine”) AND (“Suicide” OR “treatment-emergent suicidal ideation”) AND (“Young” OR “Youth”)].

**Results:**

A total of 31 manuscripts were retrieved and 6 were selected, 3 original research and 3 non-systematic reviews of randomized clinical trials. Only studies written in English that provided information about suicidality with SNRIs in young adults with MDD.

Globally, studies show that not only antidepressants decrease the risk of suicide attempt in depressed patients, but also there is no evidence of an increased suicidality in young adults treated with SNRIs.

Interestingly, one study showed that increasing suicidality could be related to side effects of the treatment, such as anxiety, agitation and irritability. The authors found that poor antidepressant response and greater severity of depression during follow-up were associated with treatment increasing suicidal ideation, as it was suggested in another study.

Another study reinforced that there may be an emotional component to the activating effects produced by some antidepressants that could explain their controversial association with rare cases of suicidal ideation and behaviour.

**Conclusions:**

In conclusion, growing evidence shows that antidepressants overall decrease the risk of suicide attempt in depressed patients. Therefore, reducing antidepressant use over the FDA concerns about increased suicidal tendencies in young patients may actually increase suicide risks due to inadequate treatment of depression. Additional studies are essential to further confirm the importance of early treatment for depression.

**Disclosure of Interest:**

None Declared

